# Therapeutics

**Published:** 1878-06

**Authors:** 


### Therapeutics.

Sodic Salicylate in Diabetes Mellitus. Ryba and Plumert. (*Präger Med. Woch*).—These authors have reached the following conclusions: 1. In daily amounts of two drachms, it determines a decided diminution in amount of sugar excreted. 2. The best results are to be derived from more recent cases. 3. The above diminution may be made more striking by restricting the hydrocarbonaceous elements of the diet. 4. The polyuria usually yields consentaneously with the glycosuria. Other diabetic symptoms (e. g. bodily weight) are favorably influenced. In two cases the symptoms were at first aggravated, but soon yielded, under this treatment.—*Dublin Jour. Med. Sci.*

Cerii Oxalas in Chronic Cough. (*Practitioner*, April, 1878.)—Dr. Thomas Clark considers this drug to be purely sedative, and therefore a great desideratum in treatment of lung diseases, inasmuch as it does not disturb the digestive tract—the only unpleasant subjective feature of its use being occasional dryness of the mouth. In gr. v. doses he has found that it will relieve many harrassing coughs, irrespective of the pathological conditions which cause them. Dyspnœa is usually relieved at the same time. He claims that relief for a period of twenty-four hours often follows a single dose taken before rising in the morning.

Glicerinum in Treatment of Internal Hæmorrhoids. Dr. G. P. Powell, (*The Practitioner*, April, 1878).—Dr. P. commends very highly the administration of glycerine in this most annoying complaint. He gives ʒj ad ʒss *ter in die*, together with a little citric acid and anodyne p. r. n.; or it may be flavored with tinct. cardamom. comp.

He leaves the explanation of its beneficial effect to others, while he is convinced that in such cases—especially the hæmorrhoids of drunkards—it is a most efficient therapeutic agent.

